# Foreign Summary

**Published:** 1838-12-19

**Authors:** 


### FOREIGN SUMMARY.

*On the Late Prevalence of Variola in London and in Paris.*—Our metropolitan readers are no doubt well aware that, during the last six or seven months, there has been an unusual prevalence of the variolous contagion in and around London; and the same has been found to be the case, we believe, in numerous districts of the country.

From a short article in a recent number of one of the French medical journals, it seems that small-pox has existed to a very considerable extent in Paris also for some months past.

In almost every hospital numerous cases were observed; and in some of the districts or *arrondissements* of the metropolis, it appears to have been unusually fatal:—in one alone there were twenty-three deaths in the course of a month.

The disease was not confined to any particular age: but old people certainly were very rarely affected with the contagion.

Many of the patients had either been vaccinated, or had passed through the small-pox (we suppose from inoculation.)

In not a few instances was the disease of a confluent character; and some cases proved very rapidly fatal from the affection of the throat and chest.

The precursory symptoms were very generally severe pains in the limbs, alternations of chills and flushings of heat, intense headache, and irritability of the stomach.

The epigastric pain, however, so much insisted upon by Sydenham as one of the most pathognomonic premonitory signs of variola, was very frequently absent.

The eruption usually made its appearance on the second or third day; often it did not cease at this period, but continued with much severity, and became now complicated with a most troublesome angina. The inflammation of the throat, and the congestion of the lungs, were certainly the most troublesome and dangerous symptoms; and hence when these were at their *acmé*, the risk was the greatest.

In all the fortunate, though severe, cases, the pustules filled well, the face was enormously swollen, the fever was high, but the respiration, however uneasy, was not seriously distressed. On the other hand, in the fatal cases, the pustules remained low and imperfectly distended, the swelling of the features was often not great; but the breathing was much quickened from the beginning, and became more and more difficult, as the disease advanced.

A few cases proved very troublesome after the dessiccation of the pustules had taken place, in consequence of the numerous boils and circumscribed abscesses making their appearance on different parts of the limbs and body.

With respect to the treatment which was found to be most useful in this epidemic of variola, the cautious employment of blood-letting in the very early stage of the disease, was certainly beneficial. It seemed to facilitate the eruption of the pustules, and also to promote their maturation.

The use of the lancet, however, in variola, always requires much discretion, as we must never forget what expenditure, so to speak, of vital energy nature has to undergo in the subsequent stages of the disease. When we are at all doubtful as to its employment, it is better to trust to the administration of emetics and nauseants to reduce any excessive pyrexia, which may be present at the beginning of the disease.

When the eruption has fairly taken place, and provided there are no unpleasant local complications present, the best therapeutic rule is *laisser faire la Nature.*

The most critical period or stage of the disease is that of the suppuration of the pustules: the danger arising either from a deficiency of vital energy, or from a feverish excitement, often connected with some local mischief in the throat, lungs, &c. of the system. Certainly one of the most useful of remedies in this stage of variola is the application of blisters to the lower extremities, either to the thighs or to the legs. The exhibition of gentle aperients, with mild vegetable tonics, is generally useful towards the close of the disease.—*Med. Chir. Rev.*, *from Bulletin Gen. de Therapeutique.*

*Vallet's new Ferruginous Pills.*—A report was made by Soubeiran to the Royal Academy of Medicine on a new preparation of iron, having for its base a carbonate of the protoxide. M. Vallet proposes to make a stable and constant preparation from this salt, heretofore remarkably unstable, from the speedy oxygenation of the carbonate. Various attempts have been made by pharmaciens to prevent this, but hitherto without success. M. Vallet dissolves the sulphate of iron and carbonate of soda in a solution of sugar, and washes the precipitate with the same in a linen bag, impregnated with syrup; he then drains the carbonate of iron obtained, and mixes it with the honey, evaporating by means of a salt water bath to the proper consistence. Thus, in the operation, the preservative sugary substance is always in contact with the ferruginous salt. The practice has been crowned with complete success; the carbonate underwent all the unfavourable changes of long manipulation, and yet it was found in the product in the same state as at the moment of formation. Each pill contains 1.3 grains of carbonate of iron, or 0.3 grains of protoxide: ten pills represent thirteen grains of carbonate, or eight grains of protoxide. The following advantages are stated to be possessed by the formula—1. The advantage of administering the medicine in constant doses, without fear of its changing in the course of use. 2. The form prevents the disgust produced by the acerb taste of salts of iron, especially because the excipient is very soluble, and secures the action of the medicinal base.

In seven chlorotic patients, aged from fourteen to twenty-five, presenting all the characteristics of the disease, the medicine was tried. The youngest had not menstruated; in the others the menstrual secretion was irregular or deficient. The dose was carried to ten pills daily. They were always taken easily by the patients. The digestive functions became improved during their use; and the developement of gas accompanying the use of the filings, or subcarbonate, never occurred.

"The courses were restored in five of these patients—they had not yet been established in the one of fourteen years, and did not return in the one we had bled.

"From one hundred to three hundred pills were administered to our patients, in from twelve days to a month, and were sufficient to produce an improvement or cure, as we have reported.

"It is as well to remark here, that the most protracted treatment did not require more than three hundred pills, or a little more than three drachms of carbonate of iron, which furnished an evident proof of the good state of the iron in the pills,—since we are forced to administer much larger doses of other ferruginous preparations.

"It results from our experience that the new preparation can be administered easily in the dose of ten pills daily; that this dose, which can be exceeded, suffices to produce the modification of the blood and of the economy which is desirable in chlorosis; that, so far from deranging the digestion, this preparation facilitates it, acts favourably upon menstruation, and the results which it affords appear to be more readily obtained than from the greater number of other ferruginous preparations."

Before concluding, we think it necessary to report two observations made by M. Vallet, since presenting his memoir, which are interesting in the medical history of chlorosis:

"M. Vallet has found a little iron in the urine of females in health. He has found a much greater proportion in the urine of a female labouring under chlorosis, before any treatment; but when the patient had made use of the pills of iron, and when the progress of the disease towards cure had been established, he observed that the iron was diminished in the urine, without the proportion having returned to the natural state. If new analyses should confirm the first observation, they would show that, in proportion as the blood becomes impoverished, one of its essential elements, iron, is eliminated by the urinary passages.

"The second observation refers to the passage of iron into the milk. M. Vallet, who did not find iron in the milk of women in health, has observed it in appreciable proportion, and by the same chemical means, in the milk of a woman placed under treatment with carbonate of iron. This fact may be of some importance in the treatment of infants at the breast; it is still a single one, and calls for new trials; yet we can assure the Academy, that MM. Henry and Chevallier have given the ferruginous preparation to she-asses, and have ascertained that the iron passed into the milk."

To conclude: chemico-pharmaceutic observation is favourable to the new preparation of M. Vallet, presented to the Academy; and medical observations, although as yet few, also promise happy results.—*Am. Journ. of Phar.*, *Oct.*, 1838.

---

*Treatment of Spermatorrhœa.*—Case 1. A young man, æt. twenty-three, suffered both bodily and mental debility from involuntary discharge of semen, every night. Antiphlogistic remedies, and subsequently tonics, were employed without any benefit. The extract of lettuce, prepared in the manner recommended by M. Caventou, was given, in doses of from two to eight grains daily. The amelioration was rapid, and the patient was completely cured in a fortnight.

Case II. A similar case was treated with like success with camphor, in the following formula:—Twenty grains of camphor dissolved in almond oil, and made into twenty pills, by means of gum arabic. One was taken every night, and every fourth day the dose was increased by one pill, until four pills were taken daily. The spermatorrhœa, which had existed during three years, and which had resisted various medicines, completely ceased in eight days after taking the above pills, which were continued eight days more, and the cure was complete.—*British and Foreign Medical Review, from Gazette des Hopitaux.* No. 52. 1838.

---

*On Chopart's Method of Amputating the Foot.* By M. Blandin.—At the sitting of the Academy on the 29th of May, M. Blandin presented a memoir on the amputation of the foot at the articulation of the os calcis and astragalus with the scaphoid and cuboid bones. This operation, so desirable in cases of diseases of the tarsal bone, has been generally objected to on account of the danger of retraction of the calcaneum; the action of the tendo-achillis being no longer counterbalanced by the tibialis and peroneous anticus, the tendons of which are divided in the operation. M. Blandin has performed the operation several times with success, and his expectations have never been disappointed by the retraction of the heel, and consequent depression of the cicatrix. Having recently had the opportunity of examining the stump in three individuals who have undergone this operation, he thus describes the anatomical disposition of the parts: "The tendons of the anterior muscles of the leg present, at the cut extremity, a very curious disposition, which has not hitherto been noticed. These tendons terminate upon the head of the astragalus and upon the cuboidal surface of the calcaneum, which have been deprived of their articulating cartilages by absorption; they there contract a very important insertion, which enables them to continue to transmit to the foot the action of the muscles to which they belong. In addition to this, by means of the cicatrix, they are continuous with those of the superficial muscles of the sole, which act upon the calcaneum by their remaining insertion; so that the anterior muscles of the leg, and those of the sole of the foot, form but a single system, the different parts of which concur in preventing the elevation of the heel, and enable the foot to have the degree of flexion necessary for the functions which it is still called upon to perform. So that we have, in fact, *digastric* muscles, the middle tendons of which are formed by those of the dorsal and plantar muscles united, whilst the opposed bellies are constituted by the fleshy fibres of the anterior muscles of the leg and the inferior of the foot. A bursa mucosa, formed of the remains of the synovial membranes of the articulation destroyed, is placed beneath the middle tendons of these anormal digastrics, and facilitates their motion upon the astragalus and calcaneum. We find these two powers opposing the retraction of the heel, which would otherwise appear inevitable: 1st, the new insertion of the anterior muscles of the leg into the calcaneum and astragalus; 2d, the continuity accidentally established by means of the cicatrix between the anterior muscles of the leg and those of the sole, giving the former an additional adhesion upon the inferior surface of the heel, and thus enabling them to counterbalance the action of the muscles of the calf." M. Blandin concludes by stating that retraction of the heel after this operation is rare, and would probably recur still more rarely if care were taken to preserve the tendons sufficiently long: he gives his decided testimony in favour of the operation.

In the discussion which followed, M. Larrey denied the general utility of Chopart's operation, and stated that he had seen the retraction of the heel so great that the patient could only walk by supporting the knee on an artificial leg. M.

Velpeau considered the retraction as of rare occurrence, but had seen two cases in which it had produced much inconvenience. M. Blandin, in reply, stated that he had performed the operation eleven times without the occurrence of this accident.—*Ib., from Bulletin de l'Academie.* June, 1838.

---

*On Rupture of the Perineum in Females.* By Professor Dieffenbach, of Berlin.—In this important paper, Professor Dieffenbach first deprecates the general practice of leaving slight ruptures of the perineum to the unaided efforts of nature. On examining the perineum after the healing of a rupture extending backwards for half an inch or a whole inch, no corresponding cicatrix is seen, but the perineum is found contracted and diminished in breadth, and the labia are drawn backwards, in such a manner as to enlarge the orifice of the vagina. In slight cases, this is of little consequence; but, when the rupture has been extensive, relaxation of the vagina probably ensues, with protrusion of its mucous coat, and frequently, also, prolapsus of the uterus. In straining at stool, the anterior wall of the rectum bulges forwards into the vagina; and, in other instances, the superior wall of the vagina and neck of the bladder sink into its cavity. The fæces are still retained, but flatus often escapes unexpectedly. But still these evils are small, compared with those which ensue in cases where the perineum, vagina, and rectum, have been torn in such a manner that the vagina and rectum form but one cavity. Professor Dieffenbach recommends, therefore, that, even in the slightest cases of rupture, the edges of the wound should be stitched together, and union sought by the first intention, in order to avoid deformity of the parts. The following cases are given in illustration:

Case I. A young woman, aged twenty-six, in giving birth to her first child, suffered from rupture of the perineum, extending backwards about an inch. The edges of the wound were cleaned, and three sutures applied. Two of the sutures were removed on the third day, and the other on the fourth. The union was complete.

Case II. The perineum of a woman, aged thirty, was torn to the extent of an inch and a half, in giving birth to her third child. Ten hours after the accident the sutures were applied, and were removed on the fourth and fifth days; complete re-union having been effected.

[Two similar cases are detailed, which we will not transcribe.)

Case V. In a young woman, aged thirty, who had led a very irregular life, there was found incipient prolapsus of the uterus; the genital organs were very much relaxed and very large, and the perineum was extremely narrow. It could not be ascertained whether this state was the consequence of simple dilatation, or whether rupture of the perineum had previously existed. The posterior edges of the labia were made raw, and and then approximated and retained in situ by means of eight sutures. Two additional sutures were employed to unite the mucous membrane of the vagina. The operation was completely successful: the perineum acquired considerable breadth, and the vagina became so narrow that, after her recovery, the patient resumed with great success her previous vocation.

Case VI. In a strong, robust young woman, aged twenty-six, in labour with her first child, the perineum, part of the vagina, and several inches of the rectum, were unfortunately torn. Six or eight hours after the accident, a suture was passed through the edges of the vaginal wound, and by means of it the edges of the wound in the posterior wall of the vagina were brought into view and united. In the same way the wound in the rectum was drawn forwards and stiched, and the parts were then allowed to resume their natural position. The perineum was then united by powerful sutures, and moderate antiphlogistic treatment pursued. The obesity of the patient was a great obstacle to the success of the operation; it succeeded so far, however, that the cavities of the vagina and rectum were separated from each other, and the power of retaining the fæces restored; but the re-union of the greater part of the perineum did not take place.

[Four other cases are given: two of them were of recent occurrence, the other two had existed for years. In the former two, the operation was completely successful; and partial success was obtained in the latter.]

The treatment pursued was in general antiphlogistic, and, during the first six, eight, or ten, days after the operation, the bowels were kept constipated by repeated doses of opium. When passage of the bowels could no longer be avoided, an elastic catheter was introduced into the rectum, and a stream of warm water, holding a small quantity of soap in solution, was thrown up; by which means the scybalous mass was broken, and passed without injuring the parts. The catheter was employed, in general, four times daily to empty the bladder.—*Ib., from Medicinische Zeitung.* No. 52. 1838.

---

*On Hemlock Baths in Cutaneous Diseases.* By Dr. Fantonetti.—M. Fantonetti has proved the good effects of hemlock baths in acute and chronic affections of the skin: he has used them with success in erythema, impetigo, psoriasis, lichen, and they have even alleviated the pains of gout. He regards the baths and lotions of the decoction or infusion as sedative, resolutive, and desiccative. This remedy acts promptly, and, used in a proper manner, never produces injurious effects. The bath is prepared either by infusing or boiling eight or ten small handfuls (*pincées*) of dried or fresh hemlock in eight or ten quarts of water, which is to be then poured into the bath, heated to the temperature of 90° or 92° Fahr. The patient should remain in the bath an hour or two, and should be surrounded by a blanket brought close around the neck, to prevent the vapour producing vertigo or pains in the head. M. F. supposes the hemlock to act from the alkaloid principle which it contains, and which explains, he asserts, the reason why the decoction and the infusion are equally efficacious. This alkaloid principle does not evaporate in the same manner as the volatile parts of aromatic plants, which are employed for similar purposes.—*Ib., from Revue Médicale.*

---

*Dislocation of all the Metatarsal Bones upon the Tarsus.* By M. MAZET.—The wheel of a heavy laden cart passed over the foot of a young man, aged nineteen. When examined, the foot appeared twisted on itself, the convexity of the curve being above and outwards. On the dorsal surface of the foot was a wound, across which was a bony projection, elevating the extensor brevis digitorum, and the tendons of the common extensors of the toes, apparently formed by the posterior extremities of the three middle metatarsal bones. External to this projection was another, but this was covered by skin. The bones of the leg were not broken, and the toes were uninjured. It being believed that several bones were bruised, the leg was amputated. The patient died of phlebitis. The amputated limb was dissected, and the following were its appearances: The most external digitation of the extensor brevis was torn; the tendon of the tibialis anticus was partially ruptured, about an inch from its termination; none of the muscles in the sole of the foot were at all torn; but the interossei, contained in the first and fourth space, were ruptured. All the metatarsal bones were dislocated from their tarsal articulations, although they were not displaced together towards the same point, and did not maintain their normal relations to one another: the second, third, and fourth were dislocated together towards the dorsal surface, so that their tarsal extremities rested on the superior surfaces of the cuneiform bones; the first and fifth were quite separated from their usual connexion, and were situated thus: the posterior extremity of the first rested on the inner side of the first cuneiform bone,—that is to say, it was dislocated inwards, carrying with it and stretching the tendon of the peroneus longus muscle; the fifth metatarsal bone, which had suffered the most considerably, was dislocated at its posterior extremity, which is not connected with the bones of the tarsus by either tendon or ligament. This bone was turned on its axis, so that its internal surface was upwards and, in addition, it was fractured about three-quarters of its length from its tarsal articulation.—*Ib., from Gazette des Hopitaux. Février*, 1828.

---

*Animal Magnetism.*—If the following report prove to be correct, (and it is said to be authentic,) we must, however humiliating it may be to our pride, withdraw all opposition to Mesmerism, and acknowledge ourselves to have been unreasonably sceptical on the occasion.

A young and hysterical female had suffered much from most painful menstruation, for some years. It was proposed to try the new agent to lull the pain of back and hypogastric region, during the first day of the dysmennorrhœa. For this purpose magnetized water was injected into the vagina, and a piece of nickel applied to the loins. The effect was almost magical. The whole of the uterine system fell into profound Mesmeric coma, which lasted twelve hours, after which menstruation went on calmly and free from pain. The same process was employed at the next catamenial period, and with equal success. The third period passed without pain, and without any menstruation at all.

This was considered to be accidental, and that, at the fourth epoch, all would come right. The fourth passed, however, in the same way as the third, and the consequence of this obstruction was, morning sickness, and some qualms and caprices as to certain articles of diet. Soon after this, the mammæ enlarged a little, and the areola round the nipples became of a darker hue. Still later the young female became plumper about the abdomen than she used to be—and, finally, there was no doubt left as to the powers of the magnetized water. The MAGNATES MAGNETICI were now in rapture, and became satisfied that the advent of a young Mesmeric Shiloah was at hand, and that HE would exhibit the zoo-magnetic powers in their highest degree of perfection. They confidently predict that he will not only have *clairvoyance* at his *finger's ends*, but that he will be able to magnetize and somnolize every living creature at the distance of the antipodes, if desirable. As our antipodes are in Australia, let the kangaroos look out sharp, or they will be "*caught napping*," and find their way into our zoo-logical gardens.

The heads of the MAGNATES are nearly turned by this event, as they prognosticate, and not without reason, that a new race of mortals—perhaps immortals—is about to appear—something of the Minerva breed—struck out of BEING'S brains, from brains struck out of BEINGS. And as successive revolutions of our earth prepared the way for successive races of animals, so the MAGNATES MAGNETICI are probably destined to introduce a new code of laws into the physical, or even moral world—the old laws of Nature and Nature's God beginning now to be effete.

But let the philosophers of the new light ponder a little on the results of their discoveries. When the modern Prometheus succeeded in infusing a spark of life into the man-monster at Geneva, he little thought that he was bringing into the world a demon over whom he would have no control; but who, on the contrary, would embitter the days of his creator, and destroy the lives of innocent victims! So the magnetizing Frankensteins may be unchaining a monster who may overwhelm the liberators in ruin or destruction.

Let us come a little closer to the question. Animal magnetism must either be true or false—a fact or a fiction. Suppose it be true—and see the consequences. By a single wave of the hand, we deprive a female of all sense, and throw her into such a profound sleep that the teeth may be pulled out of her head, without the slightest consciousness on her part. Should such a power on the one side, and such susceptibility on the other, be once established, no female in the realm, however high or low her station, would be one day safe from the machinations of the wicked and licentious! In short, the whole foundations of society would be broken up, and every fence of virtue and honour would be levelled in the dust!—*Med. Chir. Rev.*

